# The Impact of Overtraining on Injury Rates in School-Age Athletes—A Scoping Review

**DOI:** 10.3390/jcm14134712

**Published:** 2025-07-03

**Authors:** Rafał Borkowski, Justyna Krzepota, Mikołaj Wróbel, Dominika Madej, Michalina Błażkiewicz

**Affiliations:** 1 Faculty of Rehabilitation, The Józef Piłsudski University of Physical Education, 00-968 Warsaw, Poland; michalina.blazkiewicz@awf.edu.pl; 2 Institute of Physical Culture Sciences, University of Szczecin, 70-453 Szczecin, Poland; 3 Carolina Hospital Orthopaedic Department, 02-757 Warsaw, Poland; mwrobel.orto@gmail.com; 4 Faculty of Health Sciences, Medical University of Gdańsk, 80-210 Gdańsk, Poland; dominika.madej@gumed.edu.pl

**Keywords:** overtraining, physical activity, primary school children, fatigue, adolescent, training load

## Abstract

**Background/Objectives**: In developed countries, an increasing number of children are participating in sports activities. However, according to current evidence, early sport specialization and overtraining are associated with an increased risk of injuries in youth athletes. Despite the prevailing practice of early sports specialization, there is a paucity of publications summarizing the influence of overtraining on injuries in youth athletes. As a result, the objective of this study was to conduct a review of publications investigating the influence of overtraining on injury prevalence in sport-active children aged between 7 and 14 years. **Methods**: The review included publications from January 2015 to March 2025 searched via PubMed, Science-Direct, and Scopus. **Results**: This review examined five publications, across which in three papers, the authors analyzed the influence of training load on the incidence of injuries during a certain period. In one study, the authors retrospectively analyzed whether there is a relationship between training frequency during skeletal growth and femoral neck deformity in adults. The other study was a case report of reactive osteochondromatous lesions of the femoral neck due to youth athlete training. In four publications, the authors reported the negative effects, and in one, no effect of training load on youth athletes. **Conclusions**: The current evidence suggests a potential association between high training load and elevated injury risk in young athletic populations. Nonetheless, inconsistencies across studies indicate that this relationship is likely multifactorial.

## 1. Introduction

Physical activity (PA) is widely recognized as a fundamental pillar of healthy childhood development, contributing significantly to physical, mental, and emotional well-being [1]. Regular engagement in physical activity, particularly through organized sports, has been associated with a multitude of benefits. These include the promotion of musculoskeletal health [2], enhancement of cognitive function, and improved academic performance [3]. In addition, early involvement in sports can foster critical life skills, such as discipline, teamwork, and resilience.

Despite these established benefits, current data suggest a concerning trend of physical inactivity among children. According to The 2024 U.S. Report Card on Physical Activity for Children and Youth, only 20–28% of American children aged 6 to 17 meet the recommended guideline of at least 60 min of moderate to vigorous physical activity per day [4]. A similar pattern is evident across Europe, where merely 17.6% of 15-year-old boys and 9.6% of girls meet the World Health Organization (WHO) recommendations for daily physical activity [5]. Analysis of 298 population-based surveys has revealed that in the Asia Pacific region, 89% of boys and 95.6% of girls presented insufficient physical activity levels [6]. Moreover, a study on Singaporean adolescents showed that none of 233 participants met the required 60 min of daily moderate to vigorous physical activity [7]. These statistics highlight a persistent public health challenge.

Interestingly, amidst this general decline in habitual PA, a contrasting trend has emerged in developed countries: increased participation in structured and competitive youth sports [8]. This rise in organized sports involvement appears to be closely linked with socio-economic factors. Studies have shown that children from families with higher socio-economic status and parental education levels are more likely to participate in regular, organized sports programs [9,10]. This correlation may be attributed to greater access to resources, facilities, and parental support for athletic pursuits.

While this shift towards structured physical activity is promising in terms of addressing physical inactivity, it is not without its drawbacks. One primary concern is the pressure associated with competitive youth sports. The proliferation of youth sports leagues and talent development programs has amplified expectations for high performance, which can lead to high training volumes [10]. This intensification of training may exceed the adaptive capacity of growing children, whose developing physiological systems are more vulnerable to stress and fatigue [11]. Inadequate recovery in such scenarios can lead to overtraining syndrome (OTS), a condition characterized by physical exhaustion, emotional disturbances, and decreased performance [12]. OTS can be assessed through a combination of subjective indicators (e.g., mood, sleep disturbances, perceived fatigue) [13] and objective measures (e.g., hormonal markers, heart rate variability) [14]. Importantly, the recovery process is not solely about rest but also includes the strategic management of training load intensity and periodization. In children, whose bodies are still maturing, inappropriate training schedules can lead to long-term consequences, including increased susceptibility to injuries, such as stress fractures, tendinopathies, and growth plate disturbances [15].

Another critical issue in youth sports is early specialization. Defined as intensive, year-round training in a single sport (typically over 8 months per year) while excluding participation in other sports [16], early specialization has become increasingly prevalent. Research indicates that this practice may elevate the risk of overuse injuries and psychological burnout, while potentially hindering long-term athletic success [8]. Iona et al. [17] reported that 17% and 41% of youth athletes are involved in early specialization, often driven by external pressures from coaches, parents, and competitive frameworks.

While early specialization may result in early athletic proficiency and success in junior competitions, it may paradoxically be detrimental in the long run. A meta-analysis by Güllich and Barth [18] found that although participation in talent development programs correlates positively with performance at the junior level, it correlates negatively with success at the senior level. These findings underscore the importance of a diversified athletic experience during childhood as a foundation for sustained performance and physical health.

In recent years, a growing number of publications have explored the psychological implications of overtraining in young athletes, including associations with anxiety, depression, and burnout [19,20]. It should be noted that after the COVID-19 outbreak, there was a change in participation rates in youth sports. For instance, in the US [21], participation dropped from 57.4% in 2019 to 47.7% in 2021. Interestingly, the largest decline was among teenagers participating in two or more sports (31.5% in 2019, compared to 23.1% in 2021). On the other hand, in Korea [22], regular participation in sports in 2020 (52.04%) increased compared to 2019 (50.06%), but only among the adolescent group. However, it must be emphasized that COVID-19 restrictions have led to a significant decline in youth engagement in physical activity [23], have had a significant inhibitory effect on the motor development of school-aged children, and are contributing to a decrease in their overall physical fitness [24], which may result in several health consequences. Therefore, given the increasing sports specialization of children and the intense pressure to achieve a high performance at an increasingly young age, the prevention of overtraining injuries assumes special importance. An important step in this regard is the development of The Youth Overuse Injury Questionnaire tool by Lau and Mukherjee [25]. In addition, numerous published papers confirm the effectiveness of education and prevention programs for youth athletes [26,27,28], which serve to improve knowledge and early identification of risk factors for injury and indicate proposed solutions in this area [29].

However, there remains a notable gap in the literature regarding the specific relationship between overtraining, inadequate load management, and the incidence of physical injuries in children. A more focused examination of this relationship is essential for developing evidence-based guidelines to protect young athletes from avoidable harm. It is essential to distinguish between two closely related but distinct concepts in sports medicine: overtraining syndrome (OTS) and overuse injuries. Overuse injuries refer to localized musculoskeletal disorders resulting from repetitive microtrauma that occurs without sufficient time for tissue recovery. These injuries commonly manifest as tendinopathies, stress fractures, and growth plate disturbances [30]. While both overuse injuries and OTS are associated with excessive training loads, they differ significantly in their underlying mechanisms, diagnostic criteria, and clinical management. This review specifically examines the link between overtraining, particularly when training loads are excessive or poorly regulated, and the incidence of physical injuries in youth athletes. The primary objective is to synthesize current evidence regarding the influence of overtraining on injury prevalence in physically active children aged 7 to 14 years. Furthermore, this study aims to identify prevalent injury patterns, contributing risk factors, and effective prevention strategies to support coaches, parents, and healthcare professionals in promoting safe athletic development in pediatric populations.

## 2. Materials and Methods

This scoping review was not registered in the Open Science Framework or other platforms [31]. However, it was conducted and reported following the PRISMA 2020 guidelines [32,33].

### 2.1. Search Strategy

This review focused on publications examining the influence of overtraining on injury occurrence in school children. Two authors (R.B. and J.K.) independently conducted an electronic search of the PubMed, Science-Direct, and Scopus databases in March 2025. The search was limited to papers published in last 10 years, which included the following terms: children AND overtraining, adolescents AND overtraining, school children AND overtraining, children AND overtraining AND injury, adolescents AND overtraining AND injury, healthy children AND overtraining, healthy adolescent AND overtraining, overtraining AND school children NOT mental health, overtraining AND adolescents NOT mental health, overtraining AND youth sports, youth athletic performance AND overtraining, training load in youth AND injury prevention.

### 2.2. Eligibility

Only full-text peer-reviewed articles in English, published between January 2015 and March 2025, were selected from the electronic databases. The inclusion criteria were (1) studies involving human children aged between 7 and 14 years; (2) children with typical physical and mental development; (3) studies conducted on children engaged in organized sports or physical activities, such as training in clubs or participating in competitions; and (4) attribution of overtraining to injuries was defined as cases where the authors associated injuries with a high training load, repetitive microtrauma, or overuse mechanisms. Exclusion criteria were as follows: (1) publications in languages other than English; (2) conference materials; (3) reviews and meta-analyses; (4) guidelines; (5) studies involving children with chronic, congenital, or mental disorders; (6) studies reporting injuries that the authors did not attribute to overtraining; (8) studies exploring the influence of specific injury prevention programs; (9) studies including children younger than 7 years, adolescents older than 14 years or adults; and (10) studies focusing on the mental health of young athletes.

### 2.3. Review Process

Duplicate articles from different sources were removed. Titles, abstracts, and full texts of retrieved publications were sequentially reviewed by two authors (R.B. and J.K.) to determine their relevance. The reference lists of all included studies were searched manually to identify additional relevant studies. In case of doubt, M.W. resolved the issue and decided whether to include or exclude the paper in the review. The publications ultimately included in the review were checked and approved by M.B., who compiled all articles using reference management software (EndNote X7.7, Clarivate Analytics, Philadelphia, PA, USA). Due to the large heterogeneity of the included studies, a risk of bias assessment was not conducted.

### 2.4. Data Extraction and Synthesis

Data from the included studies were extracted using a predefined data extraction form developed for this review. The following information was collected from each publication: study design, characteristics of the study population (sample size, age, sex, anthropometric data), type of trained sport, study objectives, methods of training load assessment, injury and/or illness outcomes (type, frequency, severity, anatomical location), and key findings and conclusions related to the relationship between overtraining and injury risk. Discrepancies in data extraction were resolved through discussion, and, if necessary, by consulting a third author (M.W.).

Due to the heterogeneity in study designs, populations, and outcome measures, a narrative synthesis of the extracted data was performed. The results were grouped into five sections: (1) a description of the participants, (2) a summary of the aims of the studies, (3) a summary of methods and outcome measures, (4) a summary of the most commonly affected injury sites, and (5) a synthesis of the results. The findings were summarized in tabular form (Table 1) and described qualitatively in the Results section.

## 3. Results

**Figure 1:**
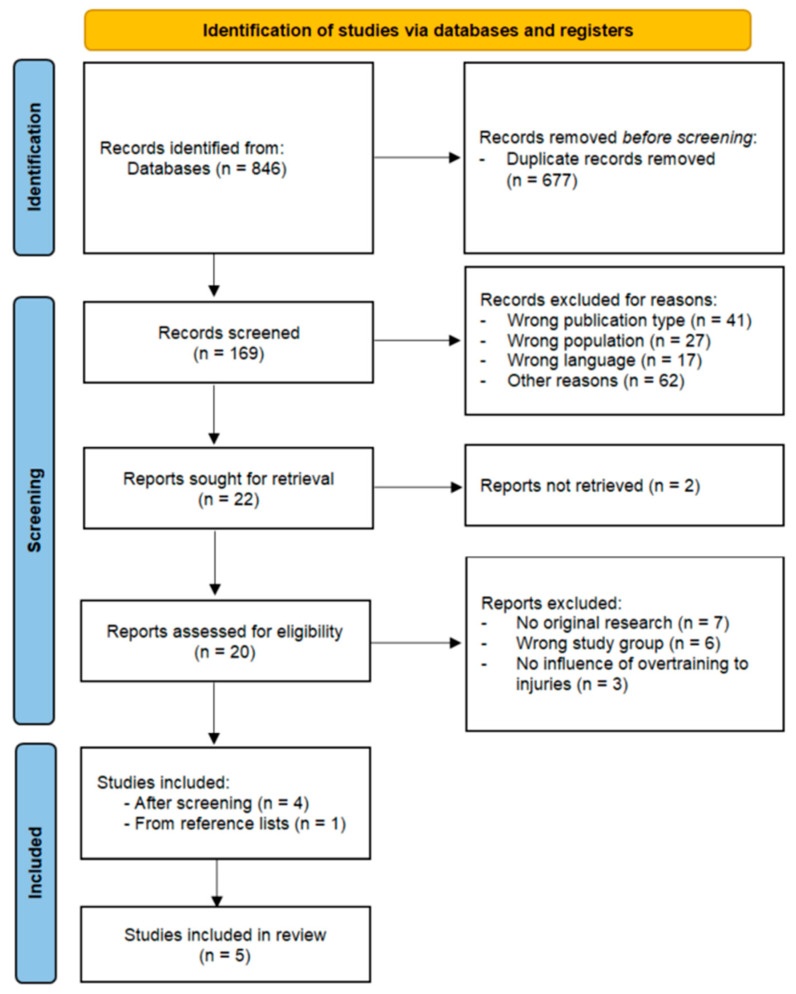
A flowchart demonstrating the selection of articles through the review process [32,33].

An initial search of electronic databases identified 846 articles. In the first stage, 677 duplicate records were removed. The remaining 169 articles were then screened based on their titles and abstracts. Of these, 147 were excluded for reasons such as an inappropriate publication type, an irrelevant study population, being written in a language other than English, and other disqualifying criteria. This left 20 articles for full-text review. Following a more detailed evaluation, 16 articles were excluded due to reasons such as lack of original research, inapplicable study groups, or failure to address the relationship between overtraining and injury incidence in children. Ultimately, four articles met all inclusion criteria. Additionally, a manual search of the reference lists of the selected studies yielded one more eligible publication. In total, five studies were included in the final review (Figure 1).

From each included publication, the following data were extracted for review: characteristics of the study population, study objectives, details of the intervention, and outcomes related to the intervention (Table 1).

**Table 1:** Summary of papers reporting the influence of overtraining in youth athletes.

Study	Study Design	Study Group: Age [years]; Body Mass [kg]; Body Height [cm]	Aim	Intervention	Affected Body Region	Results
Tak et al. [34]	Retrospective cross-sectional study	63 footballers (63M); Age: 23.1 ± 4.2; Weight: 78.2 ± 7.9; Height: 182.3 ± 7.3. Age at start of football: 6.4 ± 2.4; Age of professional football start: 12.5 ± 2.9	To investigate if there is a dose–response relationship between the frequency of playing football during skeletal growth and the presence of a cam deformity To investigate whether the age at which a player starts playing football is associated with the presence of a cam deformity in adulthood.	Cam deformity: Weight bearing in AP and frog-leg lateral radiographs. Measurement of the α angle in both hips. The threshold for cam deformity was set to α angle >60°. The threshold for pathological cam deformity was set to α angle >78°. Athletic activity: The age at which participants started playing football, the number of plays per week, and leg dominance were assessed by questionnaire.	Femoral neck	There was a higher prevalence of cam deformity in frog-leg lateral radiographs in players who started professional football training at a younger age. There was no difference in AP radiographs.
Matsuda et al. [35]	Case report	1 footballer (M) Age: 11; Weight: 39; Height: 142	To describe a bony bump on the femoral neck possibly induced by repetitive microtrauma, probably due to overtraining as a soccer goalkeeper.	X-ray, MRI, and CT were performed to find the cause of hip pain. Arthroscopy was performed to collect samples for histology and to perform resection of excessive tissue.	Femoral neck	The patient successfully returned to play within four months postoperatively, with no recurrence of symptoms observed during the one-year follow-up period.
McGowan et al. [36]	Cross-sectional study	914 children (376M); Age: 12.6 ± 0.5; Weight: nd.; Height: nd.	To investigate whether early specialization and exceeding current recommendations regarding sport participation volume are associated with injury history in children.	Questionnaire assessment of specialization, participation volume, and occurrence of sports injuries in the last 12 months.	Lower limb (62%); Upper limb (26%); Head and torso (12%).	Neither early specialization in one sport nor exceeding currently recommended sport participation volumes increased the odds of reporting a history of injury.
O’Keeffe et al. [37]	Prospective cohort study	97 Gaelic footballers (97M); Age: 13.4 ± 1.1; Weight: 59.3 ± 12.5; Height: 160 ± 10.	Impact of internal load on injury incidence in male adolescent Gaelic footballers.	A self-recall diary was utilized to record sports and recreational activities. Injuries were monitored for 15.2 ± 8.9 weeks. Injury onset, occurrence, location, nature, mechanism, and severity (days missed from participation) were recorded.	Lower limb (68%); Upper limb (23%); Trunk (9%).	Higher than average values of weekly load, monotony, and absolute load change were significant injury factors.
Hildebrandt et al. [38]	Prospective cohort study	91 elite ski racers; M: 52; Age: 12.1 ± 1.3; Weight: 42.7 ± 8.8; Height: 152.6 ± 9.8. F: 39 Age: 12.0 ± 1.3; Weight: 43.5 ± 9.7; Height: 152.4 ± 10.0.	To prospectively examine training load as it relates to the occurrence, severity, and burden of injuries and illness in youth alpine ski racers.	Prospective analysis of the training load characteristics, traumatic injuries, overuse injuries, and illness throughout 1 season.	Knee (39%); Lower leg (18%); Ankle (12%); Spine/back (10%); Upper leg (7%); Hip (5%); Foot (5%); Head (2%); Shoulder (2%).	A higher intensity and volume of the training were associated with increased illness, but not a higher risk of injury.

Abbreviations: M—male; F—female; nd.—no data; MRI—magnetic resonance imaging; CT—computed tomography.

### 3.1. Characteristics of Participants

The five studies included in the review investigated a total of 1166 participants, with sample sizes ranging from a single case report to large-scale studies involving over 900 children. Participant ages ranged from 11 to 13 years, covering early and late developmental stages relevant to youth sport. It is worth noticing that although the mean age of participants in the study by Tak et al. [23] was 23.1 ± 4.2, the retrospective data referred to children between 6.4 and 12.4 years. Most studies focused exclusively on male athletes, apart from the study by Hildebrandt et al. [38], which included both male (*n* = 52) and female (*n* = 39) elite alpine ski racers.

Body mass and height were reported in four of the five studies. Tak et al. [34] reported a mean body mass of 78.2 ± 7.9 kg and a height of 182.3 ± 7.3 cm in adult football players with a history of early training. The authors did not provide retrospective weight and height data. In contrast, younger participants, such as an 11-year-old athlete who participated in a study by Matsuda et al. [35], had a significantly lower body mass (39 kg) and height (142 cm). The study by O’Keeffe et al. [37] reported an average body mass of 59.3 ± 12.5 kg and height of 160 ± 10 cm in adolescent Gaelic footballers. Hildebrandt et al. [38] presented sex-specific data for youth ski racers, with boys and girls showing similar anthropometric characteristics (mean weight approximately 43 kg and height around 152 cm).

One study, conducted by McGowan et al. [36], involved a large cohort of 914 children (376 males) with a mean age of 12.6 ± 0.5 years, although body mass and height data were not provided. Overall, the participants represented a range of competitive youth athletes across various sports, including football (soccer), Gaelic football, and alpine skiing, offering a diverse perspective on the relationship between overtraining and injury risk in young athletes.

### 3.2. Summary of Study Aims

All five studies examined the impact of overtraining or excessive sports participation on youth athletes, though with differing emphases. Three studies [34,36,38] focused on larger groups to explore broad patterns, such as the link between training volume and injury risk or long-term musculoskeletal adaptations. In contrast, Matsuda et al. [35] presented a single case highlighting the effects of repetitive microtrauma from overtraining. While Tak et al. [34] investigated structural bone changes (cam deformity), McGowan et al. [36] and O’Keeffe et al. [37] focused on the relationship between injury risk, specialization, and internal load. Hildebrandt et al. [38] uniquely examined both injuries and illnesses in elite youth athletes across a season. Despite differing approaches, all of the studies underline the importance of managing training load in young athletes.

### 3.3. Summary of Study Methods and Outcome Measures

The reviewed studies employed a range of methodologies to assess the effects of overtraining, reflecting both clinical and observational approaches. Most studies relied on non-invasive data collection tools, such as questionnaires [34,36,37] and training diaries [38], to gather information on training history, volume, specialization, and injury occurrence. These tools enabled researchers to analyze patterns across larger youth sports populations.

Tak et al. [34] complemented questionnaire data with radiographic imaging to detect structural changes in the hip (cam deformity), linking training intensity during growth with long-term bone adaptations. Hildebrandt et al. [38] conducted a season-long prospective study, recording training loads and injuries in real time, offering a dynamic view of how training volume correlates with illness and injury risk.

In contrast, Matsuda et al. [35] reported a single clinical case, employing advanced diagnostic methods such as X-ray, MRI, CT, and arthroscopy to identify the source of hip pain and guide treatment. This approach allowed for direct pathological confirmation of training-induced injury.

Despite methodological differences, all studies shared a focus on quantifying training exposure and linking it to physical outcomes, whether through imaging, injury surveillance, or athlete self-reporting. Together, they illustrate the acute and long-term consequences of excessive or poorly managed sports training in young athletes.

### 3.4. Summary of Injury Sites

Regarding injury location, the most frequently affected regions were in the lower extremities. In the studies by McGowan et al. [36] and O’Keeffe et al. [37], injuries to the lower limbs accounted for 62% and 68% of all reported cases, respectively. Hildebrandt et al. [38] provided a more detailed distribution, identifying the knee (39%), lower leg (18%), and ankle (12%) as the most commonly injured areas among elite youth ski racers. Additionally, structural hip abnormalities were reported in football players. Tak et al. [34] found a higher prevalence of cam-type femoral head deformities in players who began professional-level training at an early age. This observation was echoed in the clinical case described by Matsuda et al. [35], where an 11-year-old football goalkeeper developed a reactive osteochondromatous lesion of the femoral neck due to repetitive mechanical overload. These findings collectively underscore the high vulnerability of the developing lower extremities to both acute and overuse injuries in the context of intensive sports participation.

### 3.5. Summary of the Results

Across the reviewed studies, a consistent theme emerged: excessive or poorly managed training in youth athletes is associated with negative health outcomes, although the specific manifestations varied. Several studies found a clear link between high training loads and increased risk of injury [34,37,38] or illness [38]. Notably, O’Keeffe et al. [37] identified that higher weekly loads and load fluctuations were significant predictors of injury, while Hildebrandt et al. [38] observed a rise in illness, but not injury, with increased training intensity and volume.

Tak et al. [34] showed that early and intensive football training during skeletal development was associated with a higher prevalence of cam-type hip deformities, suggesting a structural, long-term consequence of overtraining. Similarly, Matsuda et al. [35] described a case where repetitive microtrauma likely caused a femoral neck abnormality, requiring surgical intervention, highlighting the potential for severe individual outcomes.

In contrast, McGowan et al. [36] reported no significant association between early specialization or high participation volume and injury history in their large sample, suggesting that other moderating factors (e.g., supervision, sport type, or individual resilience) may play a role in injury risk.

Despite these differences, the overall findings support the conclusion that training volume and intensity must be carefully managed in youth athletes to prevent both acute and long-term adverse outcomes.

## 4. Discussion

This scoping review aimed to synthesize current evidence on the relationship between high training load and injury risk in youth athletes. Out of the 846 records initially identified through database searches, only five studies met the predefined inclusion criteria and were included in the final analysis. The small number of qualifying studies reflects a notable lack of high-quality, focused primary research in this field, underscoring the urgent need for further empirical investigation into how excessive training affects the health and safety of children and adolescents engaged in sports.

The included studies demonstrated substantial heterogeneity in their methodologies, including differences in study design, participant demographics, sport type, and methods of assessing training load and injury outcomes. Despite these differences, a majority of the studies reported an adverse association between elevated or poorly managed training loads and increased risk of physical injuries, primarily overuse injuries, in young athletes. It is important to emphasize that while overtraining syndrome (OTS) was not specifically assessed in these studies, high training volumes and intensities have been recognized as contributing factors to both overuse injuries and the broader clinical picture of overtraining in pediatric populations.

Two studies—O’Keeffe et al. [37] and Hildebrandt et al. [38]—identified significant associations between a high weekly training volume, training monotony, and abrupt changes in load with an increased risk of injuries and illnesses. These findings mirror patterns previously observed in adult athletic populations. For example, a systematic review by Barry et al. [39] confirmed a positive correlation between overtraining and both injury and illness among competitive adult swimmers. A meta-analysis by Franco et al. [40] demonstrated a higher prevalence of overuse injuries in adult athletes across various sports. The similarities between youth and adult athletes in response to overtraining suggest that young athletes may experience physiological stress reactions comparable to those seen in mature competitors, despite their differing developmental profiles.

Further supporting the long-term consequences of intense early sports participation, Tak et al. [34] reported a dose–response relationship between intensive football training during periods of skeletal growth and the subsequent development of cam-type femoroacetabular impingement (FAI) confirmed in adulthood. However, the development of such skeletal adaptations is likely multifactorial, as highlighted by other researchers [41]. In addition to the age at which a child begins participating in structured sports, factors such as genetic predisposition, biological maturation, cumulative training load, movement biomechanics, and the adequacy of recovery strategies may contribute significantly to the risk of injury and the emergence of musculoskeletal abnormalities. These considerations are crucial when interpreting the findings of Tak et al. [34] and contextualizing them within the broader framework of pediatric athletic development. Adding to this evidence, Matsuda et al. [35] described a clinical case involving an 11-year-old football goalkeeper who developed a reactive osteochondromatous lesion on the femoral neck, which was attributed to repetitive microtrauma from training overload. This case illustrates how localized musculoskeletal adaptations can result from sustained mechanical stress during growth. These observations align with the longitudinal findings of Fernquest et al. [42], who demonstrated that the most pronounced development of cam deformities typically occurs before the age of 14 and is strongly associated with elevated levels of physical activity during early adolescence.

Similar results were obtained by Jayanthi et al. [8], who compared a group of children and adolescents aged 7–18 years with documented sports injuries to a control group whose data came only from routine physical exams. Based on a sample of 1190 children (822 with injuries and 368 without), they found that the peak injury incidence occurred at an average age of 14.1 years, corresponding to the growth spurt period. Independent and statistically significant risk factors included the number of hours of weekly activity, the ratio of specialized training to free play, and early specialization in a single sport. Moreover, these conclusions are consistent with the clinical observations made by the authors (M.W., R.B.). In our outpatient sports injury clinic, we primarily see children aged 10–15 years who specialize in a single sport, most commonly team sports in the Polish setting (e.g., football, volleyball, basketball), and in individual disciplines like tennis. These young athletes typically have a high training volume, consisting of five training sessions and one weekly match. Moreover, they demonstrate a high degree of sport specialization with no general physical preparation activities and a narrow focus on sport-specific training. Based on clinical observations, overuse injuries predominate, the most common being Sever’s disease in younger patients, and Osgood–Schlatter disease or osteochondral lesions in the knees and ankles in older children. These are simultaneously accompanied by acute sports injuries, with ankle sprains, patellar dislocations, or meniscal and ligament injuries in the knees being the most common ones. Similar conclusions were drawn by Hall et al. [43], who analyzed a cohort of 546 female middle and high school team sport athletes (basketball, soccer, and volleyball) in terms of patellofemoral joint pain and pathology. They found that athletes who specialized in a single sport had a 1.5-fold higher incidence of patellofemoral pain (PFP) compared to those with diversified athletic activity. Moreover, the risk of mechanical injuries to the extensor mechanism (such as Sinding–Larsen–Johansson syndrome, patellar tendinopathy, or Osgood–Schlatter disease) was found to be four times higher in single-sport athletes.

In contrast, McGowan et al. [36] did not observe a statistically significant association between early sport specialization or exceeding recommended participation volumes and the likelihood of injury. This discrepancy may reflect differences in participant age, sport type, level of supervision, or training environments. It also highlights the complex and multifactorial nature of injury risk in youth athletes, suggesting that overtraining is not the sole determinant. Psychosocial stressors, biological maturation, recovery practices, and sport-specific demands likely contribute to the variability in outcomes across studies. The results highlight the importance of individualizing training programs, monitoring loads, and ensuring proper recovery periods. Coaches and parents should participate in monitoring training loads and pay particular attention to signs of fatigue in children and adolescents to prevent long-term health consequences.

However, the paucity of research on overtraining in young athletes calls for further, high-quality studies. Importantly, methodological limitations across the reviewed literature complicate direct comparisons and undermine the generalizability of findings. Studies varied widely in terms of how training load and injuries were measured. Most relied on self-reported questionnaires, athlete diaries, or coach assessments, all of which are prone to subjective bias and inconsistencies in interpretation [44]. None of the included studies incorporated objective physiological or biomechanical measures—such as heart rate variability, GPS load tracking, or neuromuscular assessments—which are increasingly recommended for accurate monitoring of training stress and recovery [45]. The lack of such tools limits the precision with which training load can be quantified and correlated with injury outcomes.

This scoping review has several limitations that should be acknowledged. Firstly, the small number of eligible studies limits the comprehensiveness of the available evidence. Notably, despite the increasing intensity of youth sports programs and growing concerns regarding overtraining in children, there remains a striking paucity of recent, high-quality research on this topic. The limited number of new studies identified over the past decade highlights a significant research gap, which restricts the ability to formulate strong, evidence-based recommendations.

Additionally, considerable methodological heterogeneity, ranging from differences in study design, population characteristics, sport types, and outcome measurement, makes it difficult to draw definitive conclusions or directly compare results across studies. Most included studies relied on subjective data collection methods, such as questionnaires and self-reported diaries, introducing the risk of recall bias and measurement inaccuracies. Furthermore, none of the studies incorporated objective physiological or biomechanical measures for training load monitoring, which could have enhanced the precision of exposure assessments. The absence of randomized controlled trials and high-quality prospective cohort studies limits the ability to infer causality.

These limitations may have affected the validity of the conclusions drawn from this review, potentially leading to overestimation or underestimation of the true relationship between overtraining and injury risk in youth athletes. Nonetheless, by explicitly adhering to a transparent review process and applying clear and strict eligibility criteria, we sought to minimize selection bias and ensure consistency in study inclusion. The findings should, therefore, be interpreted with caution and considered indicative, rather than definitive, emphasizing the urgent need for more robust, prospective research in this field.

## 5. Conclusions

In conclusion, the available literature suggests a possible association between a high training load and increased injury risk in youth sports participants. The findings of this review underline the importance of individualized training load monitoring in youth sports programs. Sports organizations, coaches, and parents should pay attention to recovery periods and avoid early specialization in order to minimize injury risk. However, inconsistencies across the existing literature indicate that this relationship is complicated and influenced by multiple variables. To inform safe and effective training practices, future research must adopt robust methodologies and aim to define evidence-based thresholds for training volume and intensity in developing athletes.
